# Characterization of Open-Cell Sponges via Magnetic Resonance and X-ray Tomography

**DOI:** 10.3390/ma14092187

**Published:** 2021-04-24

**Authors:** Gabriele M. Cimmarusti, Abhishek Shastry, Matthieu N. Boone, Veerle Cnudde, Karl Braeckman, Anju D. M. Brooker, Eric S. J. Robles, Melanie M. Britton

**Affiliations:** 1 School of Chemistry, University of Birmingham, Birmingham B15 2TT, UK; gabriele.cimmarusti@gmail.com; 2 Centre for X-ray Tomography (UGCT), Ghent University, Proeftuinstraat 86, B-9000 Gent, Belgium; shastryabhishek91@gmail.com (A.S.); Matthieu.Boone@ugent.be (M.N.B.); Verlee.Cnudde@ugent.be (V.C.); 3 Department of Physics and Astronomy, Ghent University, B-9000 Gent, Belgium; 4 PProGRess, Department of Geology, Ghent University, Krijgslaan 281/S8, B-9000 Ghent, Belgium; 5 Environmental Hydrogeology, Department of Earth Sciences, Utrecht University, Princetonlaan 8a, 3584 CB Utrecht, The Netherlands; 6 The Procter and Gamble Company, Brussel Innovation Center, 1853 Strombeek Bever, 100 Temselaan, Belgium; braeckman.k@pg.com; 7 The Procter and Gamble Company, Newcastle Innovation Center, Newcastle upon Tyne, Whitley Road, Longbenton NE12 9TS, UK; Brooker.am@pg.com (A.D.M.B.); Robles.es@pg.com (E.S.J.R.)

**Keywords:** pore size, polyurethane, open-cell foam, *T*_2_ relaxation, maximum opening, MRI, µCT, cleaning

## Abstract

The applications of polymeric sponges are varied, ranging from cleaning and filtration to medical applications. The specific properties of polymeric foams, such as pore size and connectivity, are dependent on their constituent materials and production methods. Nuclear magnetic resonance imaging (MRI) and X-ray micro-computed tomography (µCT) offer complementary information about the structure and properties of porous media. In this study, we employed MRI, in combination with µCT, to characterize the structure of polymeric open-cell foam, and to determine how it changes upon compression, µCT was used to identify the morphology of the pores within sponge plugs, extracted from polyurethane open-cell sponges. MRI *T*_2_ relaxation maps and bulk *T*_2_ relaxation times measurements were performed for 7° dH water contained within the same polyurethane foams used for µCT. Magnetic resonance and µCT measurements were conducted on both uncompressed and 60% compressed sponge plugs. Compression was achieved using a graduated sample holder with plunger. A relationship between the average *T*_2_ relaxation time and maximum opening was observed, where smaller maximum openings were found to have a shorter *T*_2_ relaxation times. It was also found that upon compression, the average maximum opening of pores decreased. Average pore size ranges of 375–632 ± 1 µm, for uncompressed plugs, and 301–473 ± 1 µm, for compressed plugs, were observed. By determining maximum opening values and *T*_2_ relaxation times, it was observed that the pore structure varies between sponges within the same production batch, as well as even with a single sponge.

## 1. Introduction

Open-cell foams are porous media with high permeability and high surface-to-volume ratios and can be produced from different polymers, such as polyurethane, polystyrene [1] and polyethylene [2]. Polyurethane (PU) foams are widely used in cleaning [3], filtration [4] and thermal insulation [5]. More recently, polyurethane foams have found applications in medical procedures, such as wound dressing [6], where they have been shown to reduce the risk of nosocomial infection and stimulate soft tissue regrowth [7]. This broad range of applications relies on the properties of these foams, such as high tensile strength [8], high liquid retention [9] and antimicrobial action [10]. Another important property of the PU foams is their compressibility, which is linked to their capacity to absorb fluids and energy, and their ability to recover from compression [11]. 

A limitation observed in applications of polyurethane foams is that they can exhibit inconsistent behaviour, even between samples from the same production batch, which is associated with variations in structural properties across the foam caused during the production process. Open-cell flexible polyurethane foams are produced by mixing polyol and diisocyanate [12]. Water is added to the mixture to cause the expansion of the foam by the formation of gaseous CO_2_, which results from the reaction between water and isocyanate [12,13]. Surfactants are often added to promote the nucleation of bubbles and pore cell stabilization, which are essential for the control of pore size and permeability within the foam [14]. During expansion, CO_2_ gas distributes unevenly within the growing bubbles, causing an anisotropic expansion of cells [15], especially along the axis of expansion. This exothermic reaction leads to variability in pore size, even between sponges produced from the same batch, which results in inconsistent behaviour in these types of foams. Thus, in order to test the performance of PU foams, for a range of applications, it is important to be able to characterise their pore network and relate this to their structure, behaviour and compressibility. 

The structure and properties of porous media have been studied extensively using a wide range of techniques, including mercury intrusion porosimetry (MIP) [16], scanning electron microscopy (SEM) [17], X-ray micro computed tomography (µCT) [18,19], nuclear magnetic resonance and magnetic resonance imaging (MRI) [20]. While MIP and SEM are widely used for studying rocks and other solid scaffolds, they have drawbacks when studying flexible or polymeric porous media, such as sponges. In the case of MIP, which is based on the intrusion of mercury within the porous medium [21], the technique requires the application of external pressures up to 10^8^ Pa. The application of such of high pressures can cause sample deformation and compression [22], which leads to inaccuracies in measurements. As a result, MIP is not suitable for flexible foams with porosities higher than 90% [21]. In the case of SEM, it has excellent spatial resolution (50 × 10^−9^ – 100 × 10^−9^ m) [23], and has been widely used to analyse the morphological properties of PU foams [24,25,26]. However, samples of PU foams need to be sectioned, which is invasive and can cause edge effects and errors in the measurements [21].

Magnetic resonance (MR) and X-ray µCT are both non-invasive techniques which offer complementary information about the structure and properties of porous media. X-ray µCT is capable of visualizing internal structures within porous objects [18], resulting in 3D images. Image contrast is based on differences in X-ray attenuation coefficient, which depends on both the chemical composition and density of constituent materials. In single-material objects, such as the foams investigated here, the 3D volume can be considered as a distribution of local density. Within certain boundaries, the spatial resolution is largely limited by the discretization of the 3D volume, hence it is restricted by the sample size. Selecting the appropriate resolution for imaging a porous object is a compromise between the pore size and representative element volume for the specific object of interest [27]. From 3D images, it is possible to extract quantitative parameters including density, porosity and pore size [28,29,30]. Recent developments in X-ray µCT have allowed the characterisation of internal and external dimensions in 3D printed parts [31] and the evaluation of flow processes in porous media filled with water and oil [32,33]. While offering valuable opportunities to visualize pore geometries non-invasively, there are several factors which can affect µCT images that are still poorly understood [34]. For instance, the selection of X-ray source is a critical factor, and inappropriate selection can cause the emission of secondary radiation, leading to reduced image contrast and ghost images. Furthermore, a major challenge for studying flow processes in porous media, such as polyurethane foams, is related to the small difference in contrast between fluid and the low-density porous substrate. Hence, there is a need for contrast agents to be added to the fluid [33]. 

MRI has typically lower spatial resolution than X-ray µCT, usually in the order of 10^–5^–10^−2^ m, and probes the 3D structure of a porous medium indirectly, through measurement of the signal of fluid contained within [35,36,37,38]. MRI is able to image the fluid within porous media without the need for additional contrast agents [39]. Moreover, the measurement of NMR relaxation times and diffusion coefficients for fluid within a porous medium is able to provide information on the structure of the pore network, including pore size distribution, porosity and permeability [36,37,38,39]. In particular, *T*_2_ NMR relaxation times are influenced by surface relaxation, especially in the presence of paramagnetic ions on the surface of the pore matrix, reduced mobility when molecules temporarily adsorb on to the pore surface [40], and internal magnetic field inhomogeneities (***B****_i_*) produced by magnetic susceptibility differences (Δχ) between the solid matrix and fluid [41]. In each of these situations, the *T*_2_ relaxation time of fluid within a pore is reduced, compared to bulk fluid, and is sensitive to the size of the pore. Hence, for a given porous medium, it is generally found that the smaller the pore, the shorter the *T*_2_ relaxation time. Thus, by knowing the relationship between *T*_2_ relaxation time and pore size, it is possible to determine the distribution of pore sizes within a porous medium [42]. In the case of rocks, it is found that the presence of a variety of paramagnetic ions and the large difference in magnetic susceptibility between matrix and fluids leads to a strong correlation between pore size and *T*_2_ relaxation time, and this has been extensively studied [43,44,45]. However, no MRI studies using *T*_2_ relaxation times have been undertaken to characterise polymeric porous systems. This is because, in contrast to rocks, polymeric porous systems, such as polyurethane foam, do not contain paramagnetic ions, and the difference in magnetic susceptibility between the porous matrix and the fluid within is low. 

Flow MRI has been used to characterise the pore structure of PU foams [46] at three different compressions. In this study, the flow of water was used to evaluate the pore structure and hydrodynamics within the 3D pore network in the foam, and how these changed upon compression. However, flow MRI was not able to directly determine, or map, pore sizes, and a pore portioning algorithm was required to characterise the microstructure of the foam and connect this to pore hydrodynamics, while flow MRI has been used on polyurethane sponges,

While these previous studies of PU foams have demonstrated the capability of both MRI and µ-CT to non-invasively characterise pore networks within PU foams, the reported methods are not able to characterise PU foams at speed or in large quantities. Such capabilities are, however, important for understanding how PU foams perform in different applications and, in particular, enable a better understanding of observed inconsistencies in results, which may be linked to variabilities in foam structure. In order to achieve this, it is necessary to develop characterisation methods which do not rely on techniques involving instrumentation found in specialist laboratories or detailed sample-by-sample analysis. It is also important that they can be performed quickly, having the potential for high-throughput analysis, and can provide a direct measure of pore size. One method of this kind, that relies on the use of *T*_2_ NMR relaxation times, is well-logging [47], which has been employed in the petrochemical industry to characterise porous rocks in oil-field boreholes. However, in order to determine whether an analogous method can be applied to characterising PU foams, it is first essential to ascertain whether *T*_2_ NMR relaxation times are able to characterise these types of samples.

In this study, we investigate the capability of *T*_2_ NMR relaxation times to characterise PU foams. High-resolution X-ray µCT of dry foam samples and ^1^H *T*_2_ NMR relaxation time of water, within the same foam samples, were acquired to characterize the structure of open-cell polyurethane foams. These experiments were performed on both uncompressed and 60% compressed samples. Pore size distributions were evaluated, for uncompressed and compressed sponge samples, by X-ray µCT and compared with *T*_2_ NMR relaxation times of water, acquired from both MR images and bulk measurements. The relationship between maximum opening, for pores within each foam sample, and ^1^H *T*_2_ NMR relaxation times, for water within the pores, was investigated.

## 2. Materials and Methods

**Figure 1:**
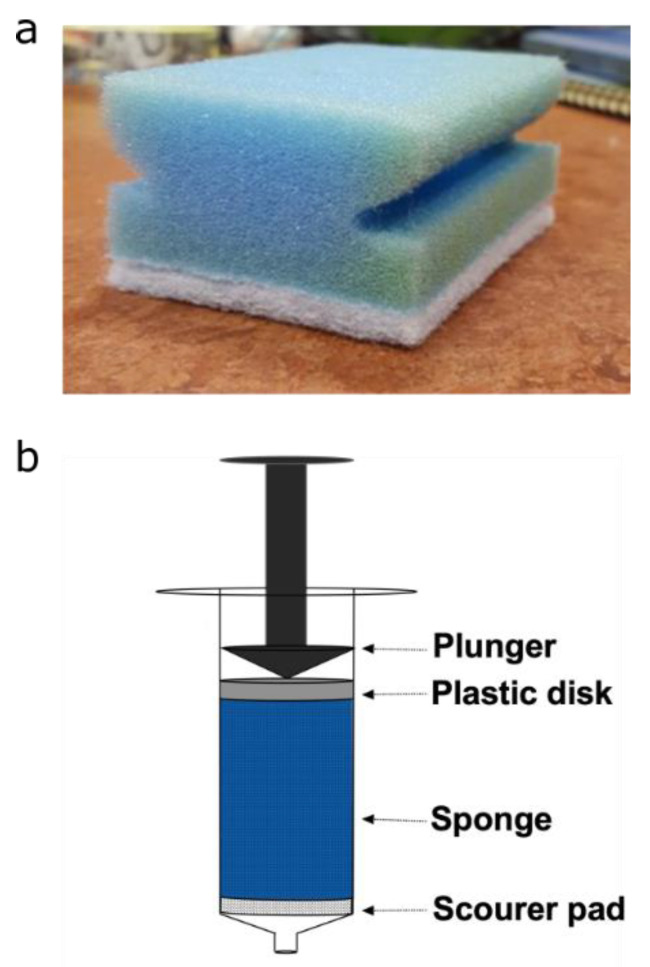
(**a**) Photograph of polyurethane sponge. (**b**) Schematic diagram for the set-up used to hold and compress sponge plugs.

X-ray µCT and magnetic resonance experiments were conducted on plugs extracted from polyurethane sponges (Spontex, Colombes, France, Batch n° = 7124813). The cylindrical plugs, of diameter 15 mm and height 40 mm, were extracted from three sponges using a 15 mm corkborer (Fisher scientific UK Ltd., Loughborough, UK). Each plug was placed into a 10 mL Terumo® syringe (Terumo®, Binan, Philippines) to position the sponges in the MRI and CT instruments, as well as to control their compression. A plastic disk of 15 mm was also placed in the syringe, at the top of the sponge, as seen in Figure 1, to enable homogeneous compression. Each plug was reproducibly compressed by adjusting the plunger in the syringe to a fixed position, resulting in 60% compression. Care was taken to ensure there was no tilting of the plastic disk during compression, leading to non-uniform compression across the plug. For the magnetic resonance measurements, plugs were saturated with 7° dH water. Air bubbles were removed by submerging the plugs under water and placing them under vacuum for one hour. All data were from the PU foam (blue part of the sponge), and the scouring pad (white part of the sponge) was not included. 

### 2.1. X-ray Computed Tomography

**Figure 2:**
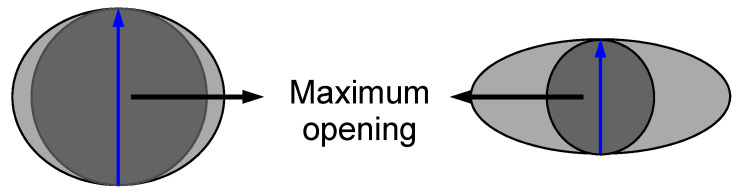
Schematic diagram showing two different pores and the spheres (dark grey) used to evaluate the maximum opening (blue arrow).

Data were acquired on the High-Energy CT System Optimized for Research (HECTOR) [48] at the Centre for X-ray Tomography (UGCT) at Ghent University, Belgium. This system consists of an XWT240 X-ray tube (X-ray WorkX, Garbsen, Germany) and a PerkinElmer flat-panel detector. For each scan, 2401 projections, of 1 s exposure time per projection, were acquired over the full 360° rotation. The tube was set at a voltage of 70 kV and a target power of 20 W, with 0.5 mm Al filter on the X-ray source to reduce beam hardening effects. Reconstruction of radiographs was conducted using Octopus reconstruction [49]. The reconstructed data had a voxel size of 40 × 40 × 40 µm^3^. Octopus analysis [30] was used to evaluate the morphology of each plug. The volume of the sponge plug was segmented by grey-value thresholding to obtain quantitative information. Maximum opening was used as an indirect measure of the size pores (Figure 2), where the maximum opening is defined as the diameter of the biggest sphere that can fit inside that pore. The distributions were normalized for the number of pores in each plug. The 3D rendering of the plugs was made using VGStudio MAX 3.2 (Volume Graphics GmbH, Heidelberg, Germany).

### 2.2. Magnetic Resonance

Magnetic resonance data were collected on a Bruker Avance III HD spectrometer (Bruker (UK) Ltd, Coventry, UK), equipped with a 7 T vertical wide-bore superconducting magnet, operating at a proton resonance frequency of 300.13 MHz. All experiments were recorded using a micro2.5 imaging probe at 293 ± 0.3 K which was maintained by the temperature of water-cooled gradient coils. ^1^H MR images were collected using a 25 mm quadrature ^1^H radio frequency (RF) WB40 birdcage coil. Bulk *T*_2_ MR relaxation measurements were collected using a 25 mm ^1^H/^19^F radio frequency (RF) WB40 birdcage coil. Sagittal (vertical) two-dimensional (2D) *T*_2_ MR relaxation images of water within the plugs were acquired using the spin echo imaging sequence RARE (Rapid Acquisition with Relaxation Enhancement) [50]. Images were recorded using 1 mm slice thickness and a 64 × 128 pixel matrix, with a field of view of 25 mm × 50 mm. *T*_2_ MR relaxation maps were produced from eight echo images, with a RARE factor of 128, echo time of 8 ms and repetition time of 15 s. A Carr–Purcell–Meiboom–Gill (CPMG) pulse sequence was employed for bulk *T*_2_ measurements, collecting 64 spectra, varying the number of echoes from 0 to 2048, logarithmically spaced, with an echo time of 2 ms and repetition time of 15 s and a spectral width of 100,000 Hz.

All MR data were analysed using the software package *Prospa* (version 3.1, Magritek, Wellington, New Zealand) [51]. A correction was required for the MR *T*_2_ relaxation maps, to compensate for the effects of diffusion, which were found to be sensitive to both the RARE factor and *T*_2_ relaxation time of the liquid [52]. Details of the calibration used can be found in Appendix A. Averaged *T*_2_ relaxation times were determined from bulk CPMG data by fitting to a single exponential, or where a single exponential would not fit the data, a double exponential. The data were fitted to a minimum number of *T*_2_ time constants. *T*_2_ relaxation time distributions (G(*T*_2_) vs. log(*T*_2_)) were determined from the CPMG data using a 1D non-negative least square algorithm (NNLS 1D) [53], where G(*T*_2_) is the distribution function with respect to *T*_2_. A regulation parameter, α, was used to assess the smoothness of G(*T*_2_) [53]. The ideal value of α was estimated by calculating the fit error (χ^2^) as a function of α. The lowest value of α was chosen before a rapid increase in χ^2^, which resulted in the narrowest distributions, without introducing spurious peaks from fitting to the noise [54]. 

### 2.3. Statistical Analysis

Scatter plots of X-ray µCT maximum opening versus bulk *T*_2_ relaxation time, or MRI *T*_2_ relaxation time, were created. Linear regression analysis was performed on both sets of data to evaluate the correlation between MR *T*_2_ relaxation times and maximum opening. Points with a standard residual close to or lower than −2 were considered as outliers [55]. SPSS version 25 was used for statistical analysis [56], and the statistical significance (p) was set as *p* < 0.05.

## 3. Results

**Figure 3:**
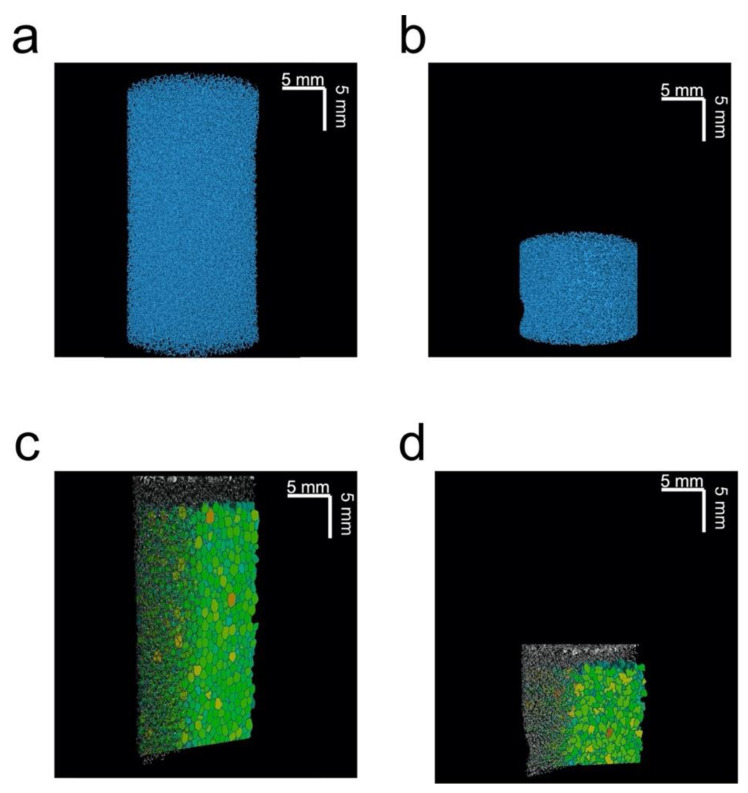
Three-dimensional µCT renderings of an (**a**) uncompressed and (**b**) 60% compressed plug. Three-dimensional renderings of a vertical clipping plane, with the maximum opening distribution for (**c**) uncompressed and (**d**) 60% compressed plug. All images are for the same plug.

Figure 3 shows 3D-rendered X-ray µCT images for a single uncompressed and c, composed of polymeric struts (blue) and void (black). Figure 3b also shows that the compression of the sponge causes some pores to collapse, while others elongate. The change in pore shape can be more easily observed in Figure 3c,d, which show sections of maximum opening for the compressed and uncompressed plug, for a plane through the centre of the sponge. From analysis of the data of the full 3D maximum opening data, it was observed that when evaluating the maximum opening for compressed plugs, the volumes of the smaller pores reduced to almost zero, making it reasonable to claim that there simply is no pore anymore. This is supported by the data shown in Table 1, which reports the number of pores for the uncompressed and compressed plug. Here, the number of pores was found to be lower for the compressed plug.

**Figure 4:**
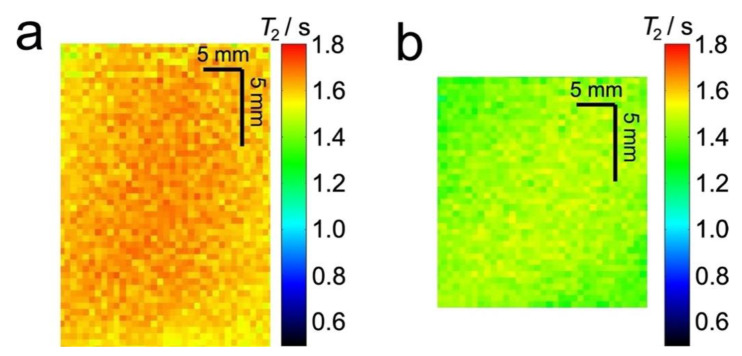
Extracted sections from 2D ^1^H MR *T*_2_ relaxation maps of 7° dH water in an uncompressed (**a**) and 60% compressed (**b**) sponge plug. Both images are for the same plug.

Figure 4 shows *T*_2_ relaxation maps for water in the same plug as Figure 3. These images show a decrease in *T*_2_ relaxation time when the sponge is compressed (Figure 4a) compared to the uncompressed sponge (Figure 4b).

**Figure 5:**
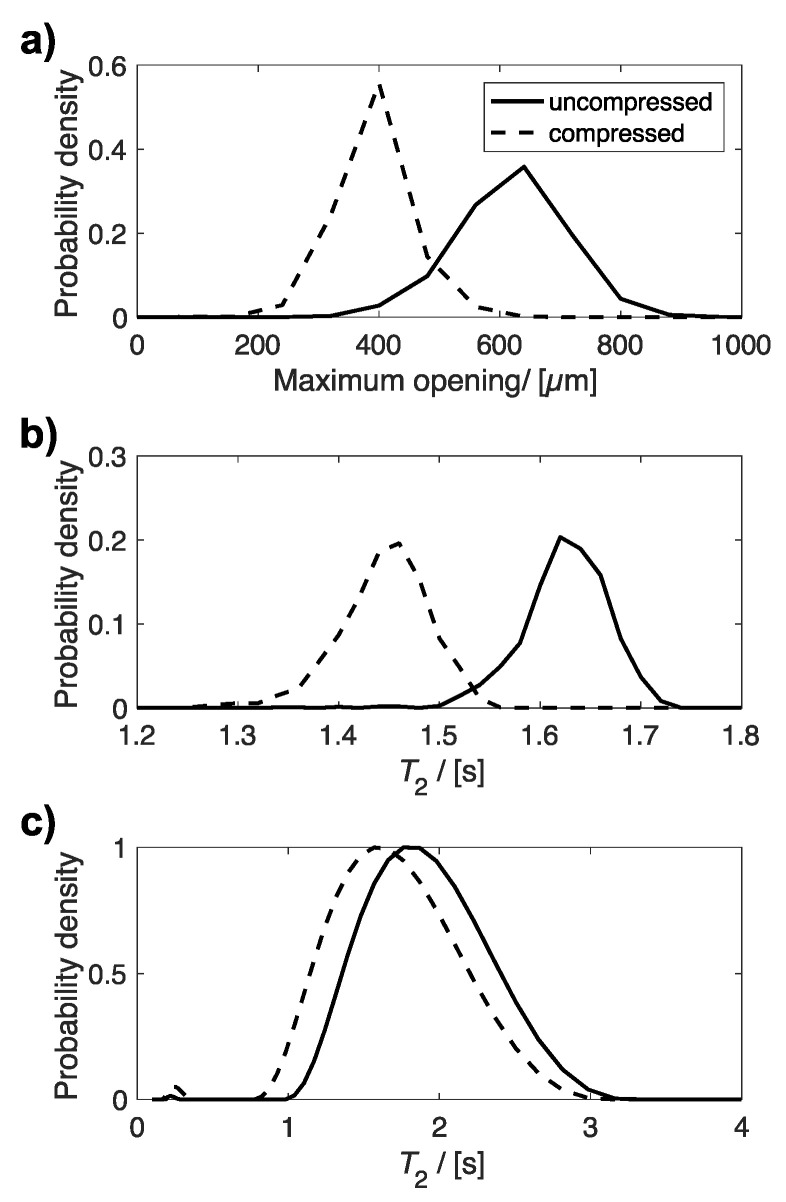
Plots of the distribution of (**a**) maximum opening, (**b**) *T*_2_ relaxation time determined from *T*_2_ relaxation MR maps, and (**c**) bulk *T*_2_ relaxation time for the same uncompressed and compressed plug.

Figure 5 compares the distributions of maximum opening (Figure 5a), determined by X-ray µCT, and *T*_2_ relaxation times (Figure 5b,c), determined from MRI and bulk NMR measurements, for the same uncompressed and 60% compressed plug. All three distributions indicate an average smaller maximum opening and shorter *T*_2_ relaxation time for compressed sponge plugs compared to uncompressed plugs. The average maximum opening and *T*_2_ relaxation times for these distributions are given in Table 2.

**Figure 6:**
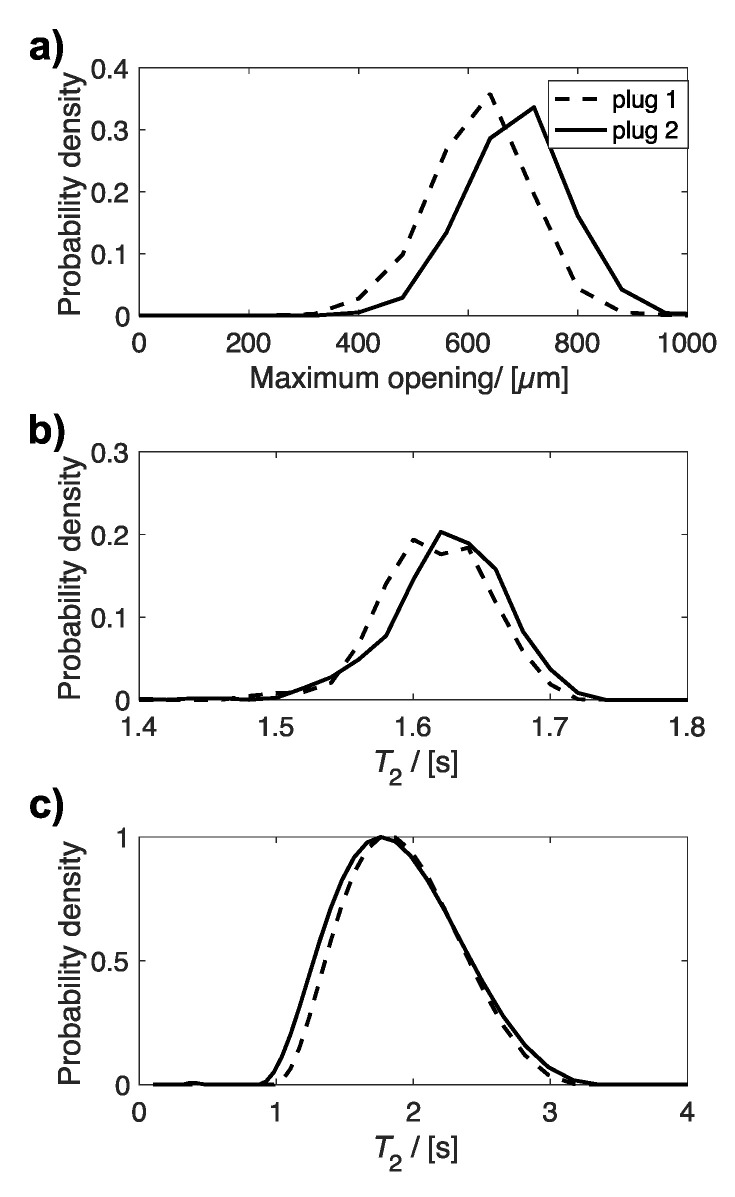
Plots of the distribution of (**a**) maximum opening, (**b**) *T*_2_ relaxation time, determined from MR *T*_2_ maps, and (**c**) bulk *T*_2_ relaxation times for two different uncompressed plugs.

**Figure 7:**
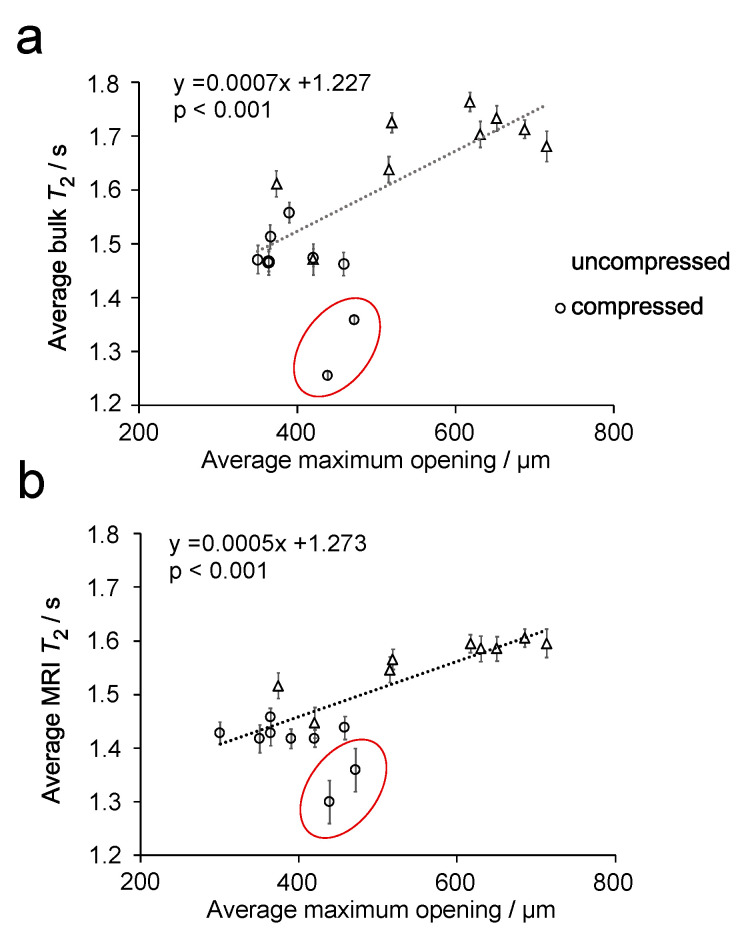
Plot of the average maximum opening vs. bulk *T*_2_ MR relaxation time (**a**) and average *T*_2_ relaxation time determined from *T*_2_ relaxation MR maps (**b**) for 7° dH water in uncompressed and compressed sponge plugs. The dotted lines are a linear fit of the data. Two outlying samples are circled in each plot.

Figure 6 compares the distributions of µCT maximum opening (Figure 6a) and *T*_2_ MR relaxation times (Figure 6b,c) for two different uncompressed plugs, which were extracted from different sponges within the same batch. The distributions for maximum opening and MRI *T*_2_ relaxation times indicate a difference between the two plugs. However, this is not observed in the bulk *T*_2_ relaxation time distributions, determined by non-linear least squares analysis. When these data were evaluated by fitting to a single exponential function, only a small difference is observed between the two plugs, with average values of 1.76 (plug 1) and 1.71 s (plug 2) determined. The relationship between average maximum opening with average *T_2_* relaxation time, determined from MRI (Figure 7a) and bulk NMR measurements (Figure 7b), are plotted in Figure 7, for all uncompressed and compressed plugs.

**Figure 8:**
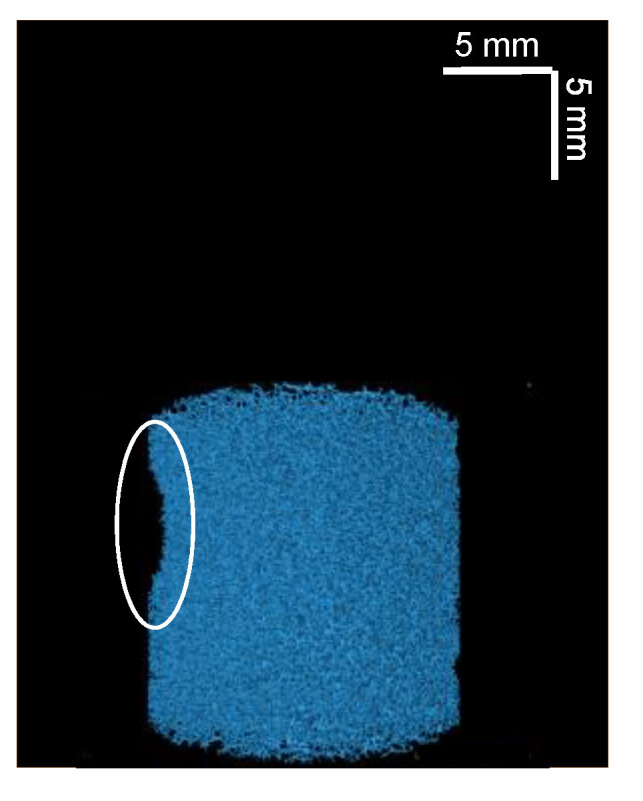
Three-dimensional µCT reconstructed image of one of the two 60% compressed plugs related to the outlier points in Figure 7. An indentation, caused by a bending of the plug when compressed, is highlighted by a white circle.

It can be seen that, for both plots in Figure 7, there appears to be a linear relationship between the maximum opening and the *T*_2_ relaxation time, where *T*_2_ relaxation time decreases with decreasing maximum opening. Statistical analysis of these data finds a *p*-value < 0.001 for both sets of data, which indicates a correlation between these parameters. In both plots, two outlying points can be observed. Linear regression analysis of these points in Figure 7, using standardized residuals, were found to be ≥−2 for the two outliers in Figure 7a,b. Hence, these points were confirmed as outliers and were not included in the fitting of the data. The outlier points were from two compressed sponge plugs and are believed to be related to a bending of the plug when compressed. This deformation can be observed in Figure 8.

## 4. Discussion

Our results across multiple sponges and compressions reveal a correlation between pore maximum opening and *T*_2_ relaxation time for fluid with pores. From close inspection of the X-ray µCT images in Figure 3, it can be observed that, when compressed, some pores collapse while others are elongated. This observation is confirmed by the decrease in the average maximum opening. However, it appears that the maximum opening tends to overestimate the actual real pore size of single pores in compressed plugs. Despite this, maximum opening can still be considered a reliable measure for sponge compression, because it represents a local, rather than a single-pore, characteristic. We found that maximum opening correlates with the *T*_2_ relaxation time for fluid within the pore network (Figure 7) and correlates better than pore size (equivalent diameter). This is possibly the case because MRI is not able to distinguish single pores, because of it has lower spatial resolution, compared to X-ray µCT. Thus, *T*_2_ relaxation times also represent a local property rather than a single-pore characteristic, which is comparable with maximum opening. While the characterisation of the pores is limited by the resolution, and image processing modalities (segmentation), the resolution at which scans were acquired was sufficient to visualise the pores of the sponge both at uncompressed and 60% compressed conditions.

Comparison of maximum opening values between different foam plugs also revealed a significant variability in pore dimensions between samples within the same production batch, and even within the same sponge. In our experiments, plugs were extracted from three different sponges within the same production batch. The variation in average maximum opening between uncompressed plugs was observed to be 422 ± 79 to 633 ± 97 µm (sponge 1), 375 ± 59 to 688 ± 96 µm (sponge 2) and 520 ± 87 to 716 ± 100 µm (sponge 3). This shows that there is variability in the average maximum opening between sponges. This demonstrated the heterogeneity in pore dimensions within the polyurethane block from which the sponges were cut.

Linear regression analysis of the data in Figure 7 demonstrates a positive linear correlation between bulk and MRI *T*_2_ relaxation time (coefficient of determination (R^2^) = 0.868, *p* < 0.001 and R^2^ = 0.915, *p* < 0.001, respectively), with the maximum opening of the pore. In particular, Figure 7 shows that as the pore decreases in size, there is a corresponding reduction in *T*_2_ relaxation time. While this effect has been observed in rock samples, it has not been previously observed in polymeric foams. Moreover, this relationship is found to be much more subtle in PU foams than in rocks, which is to be expected given the lack of paramagnetic species and small difference in magnetic susceptibility.

## 5. Conclusions

In this paper, we have shown that despite the similar magnetic susceptibilities between water and polyurethane, ^1^H magnetic resonance measurements of *T*_2_ relaxation time are sensitive to pore sizes within PU open-cell foams. Plots of maximum opening, determined by X-ray µCT, vs. *T*_2_ MR relaxation time are found to have a linear correlation. We have observed, using both X-ray µCT and MR relaxation times, that when a sponge is compressed, there is a decrease in pore dimensions. This study shows that it is possible to quantify the average pore size distribution directly from NMR *T*_2_ relaxation times, which can be mapped using MRI. Moreover, it is also possible to quantify the distribution using NMR bulk measurements, which paves the way for rapid in-line and in operando characterization of foams. These X-ray µCT and MR measurements have also demonstrated that it is possible to characterise the pore dimensions across different sponges, showing the variability resulting from the manufacturing process. We believe that such measurements will be of benefit to those studying the performance and behaviour of polyurethane open-cell foams in a variety of applications, where there is a link between pore size distribution and performance of the sponge or products within the sponge.

## Figures and Tables

**Table 1:** Total number of pores and average sphericity for an uncompressed and compressed plug from X-ray µCT.

Plug	Number of Pores
Uncompressed plug	9135
60% compressed plug	3051

**Table 2:** Average maximum opening from µCT, average MR *T*_2_ relaxation time from *T*_2_ relaxation MR map and average bulk *T*_2_ relaxation time determined from a mono-exponential fit to the CPMG data. The variability in average maximum opening and *T*_2_ relaxation time from MR maps, comes from the standard deviation of values across the sample.

Plug	Average Maximum Opening/µm	Average *T*_2_ from MRI/s	Average Bulk *T*_2_/s
Uncompressed plug	620 ± 92	1.63 ± 0.04	1.76 ± 0.01
60% compressed plug	391 ± 63	1.45 ± 0.04	1.55 ± 0.02

**Figure A1:**
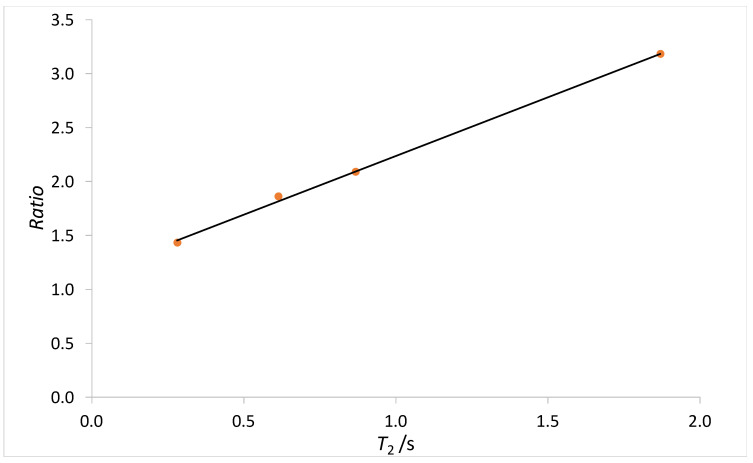
Plot of the ratio (*R*), between the *T*_2_ relaxation time obtained from CPMG and RARE experiments, vs. *T*_2_ obtained from RARE sequence. The black line represents a linear fit of the data.

## Data Availability

The data generated in this study are available at https://doi.org/10.25500/edata.bham.00000648 (accessed on 21 April 2021).

## Appendix A

The *T*_2_ of a sample recorded using RARE imaging sequence is generally affected by signal attenuation due to water self-diffusion, which is caused by the application in the read direction of a gradient (*g*_R_). Thus, the *T*_2_ observed contains a diffusion weighted component that needs to be correct. In order to evaluate the correction factor for each sample, four CuSO_4_ solutions at known concentrations (0, 1, 2, and 5 M) were been prepared. For the preparation, 7° dH water was used. Each solution was transferred in a 5 mm NMR tube. The NMR relaxation time data and magnetic resonance imaging acquisition parameter used were the same as those used for the study of water in sponge, and can be found in Section 2.3. For each sample, a ratio (*R*) between the *T*_2_ relaxation time obtained from CPMG and RARE was evaluated and plotted against *T*_2_. The plot is shown in Figure A1, which highlights that the ratio is not constant but is dependent on the *T*_2_ relaxation time.
